# Implant-Retained Prosthetic Rehabilitation of a Patient with Severe Oral Lichen Sclerosus

**DOI:** 10.1155/2023/6681076

**Published:** 2023-12-23

**Authors:** Tim Kluenter, Milan Stoilov

**Affiliations:** ^1^Department of Oral, Maxillofacial and Plastic Surgery, Medical Faculty, University of Bonn, Bonn, Germany; ^2^Department of Prosthodontics, Preclinical Education and Dental Materials Science, Medical Faculty, University of Bonn, Bonn, Germany

## Abstract

**Background:**

Lichen sclerosus is a rare condition that occurs in the genital area or on the extraoral skin but can also manifest orally (oral lichen sclerosus (OLS)). The condition is associated with atrophy, scarring, and functional limitations of the tissues. In the present case, an extremely pronounced variant is described, and the oral rehabilitation of the patient is presented. *Case Report*. The edentulous patient showed a progressive course with severely restricted mouth opening and persistent pain. Conventional dental treatment was therefore impossible. To address this issue, two implants were placed in each jaw at the position of the lateral incisors. After osseointegration and exposure of the implants, provisional bridges made of polymethyl-methacrylate were fitted to test the new jaw relation. After a successful provisional phase, an FDP in the sense of an extreme short dental arch (ESDA) has been inserted.

**Conclusion:**

The experimental treatment of the patient with only a total of four implants and the ESDA concept represented a satisfactory therapy for the patient. The patient regained her chewing ability, which significantly increased her oral health-related quality of life (OHRQoL). Still, it should be noted that this is a high-risk and experimental prosthetic treatment.

## 1. Introduction

The lichen sclerosus is a rare chronic inflammatory mucocutaneous disease of uncertain etiology that usually affects the genital area or extraoral skin but may occasionally manifest orally. Symptoms can range from simple whitish changes that cannot be wiped off to mark hardening of the oral soft tissues, accompanied by discomfort. The literature describes lichen sclerosus as a disease that can cause atrophy, scarring, and functional impairment [1]. In severe cases, the disease may lead to degeneration of elastin and collagen, resulting in hyalinization of the connective tissue and eosinophilic sclerosis.

Although it is more commonly seen in females, both prepubertal and postmenopausal, it can also occur in males [2]. The ratio of males to females affected by the condition varies between 1 : 3 and 1 : 10, with equal distribution being rare [3, 4]. While lichen sclerosus is commonly diagnosed in postmenopausal women, it may occur in approximately 50% of affected women before reaching menopause [3–6].

While there is a suggested genetic predisposition, the precise cause of lichen sclerosus is still unknown. It has been observed that around 10% of lichen sclerosus patients have relatives affected by the disease [3, 7, 8]. Immunological changes involving T and B cells have been observed in cases of vulvar lichen sclerosus, suggesting an autoimmune phenotype. These changes include elevated levels of Th1-specific cytokines, dense T cell infiltration, increased expression of BIC/miR-155, and the presence of autoantibodies against extracellular matrix protein 1 and BP180 antigen [3, 9–11]. These findings collectively point towards an immune-mediated mechanism in the development of vulvar lichen sclerosus. However, the pathogenic significance of these findings is not clear. Oxidative DNA damage and TP53 mutations (tumor suppressor gene) have also been reported, which could suggest an autoimmune basis for lichen sclerosus [3]. Pathogens such as the Epstein–Barr virus and Borrelia burgdorferi have also been discussed as causes [12, 13].

To date, only 41 histologically confirmed cases of the oral form have been reported in the literature [14]. This case report describes an extremely distinctive variant of lichen sclerosus, with a particular focus on the prosthetic rehabilitation of an edentulous patient.

## 2. Case Presentation

Informed consent was obtained from the patient at the University Hospital of Bonn, Bonn, Germany. Pertinent electronic medical records were retrospectively reviewed, including clinical documentation, radiological imaging, and photo documentation.

In April 2021, a 76-year-old female patient presented herself to our department with a request for oral rehabilitation. She had been diagnosed with lichen sclerosus in 2010, and since then, her condition had been slowly progressing, with a severe increase in mouth opening limitation. At the time of presentation, her residual mouth opening measured 35 mm from jaw ridge to jaw ridge. The opening from vermilion to vermilion and in between the oral commissures measured only 25 mm × 30 mm. As a result, access to the oral cavity was severely limited, and conventional treatment in accordance with prosthetic guidelines seemed impossible. In 2011, a surgical procedure was performed at another location involving split skin in the cheek area on both sides to address mouth opening restriction. Unfortunately, this procedure showed no improvement; on the contrary, the patient reported further progression of the symptoms. Furthermore, the patient vehemently declined any further invasive surgical intervention and expressed a preference for prosthetic rehabilitation only.

**Figure 1:**
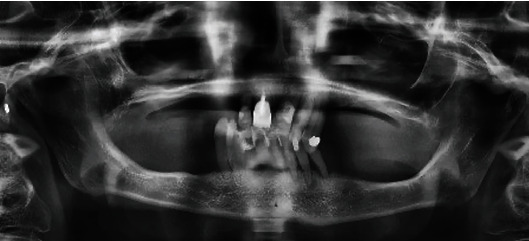
Panoramic radiograph (OPG) image depicting the patient's residual dentition not worthy of preservation and a severe generalized horizontal bone loss of 80-90%.

The remaining dentition did not appear worth preserving due to severe periodontal destruction (stage IV, grade C) (Figure 1), and the patient complained of great difficulty in ingesting food and persistent pain.

In the first step, under endotracheal tube intubation general anesthesia (ETGA), the residual dentition (Figure 1) was removed. Subsequently, the wound healed properly, and interdisciplinary planning was carried out for the rehabilitation of the patient in collaboration with maxillofacial surgery and prosthodontics. Due to the patient's mouth opening limitation and the significant hardening of the cheeks' soft tissues, particularly the outer skin and the intraoral mucosa and its dryness, a conventional total prosthesis was not feasible.

The extension of the denture had to be limited to the anterior part of the alveolar ridge since the patient's mouth opening was severely restricted both vertically and horizontally. Even the slightest opening of the mouth repeatedly led to bleeding in the area of the oral commissures and caused pain for the patient.

**Figure 2:**
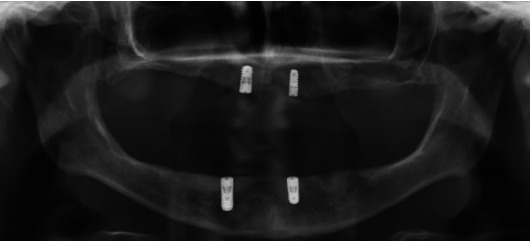
Panoramic radiograph (OPG) after implant placement and exposure at the time of impression taking. The correct fit of the scan bodies was ensured with an X-ray.

**Figure 3:**
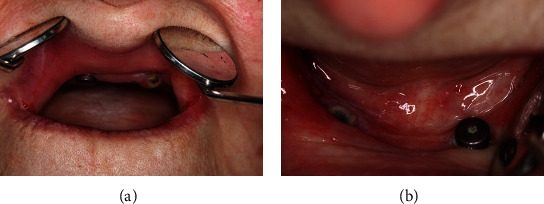
Straumann© RC (3 mm) healing abutments were used to form the implant surrounding gingiva: (a) upper jaw and (b) lower jaw.

**Figure 4:**
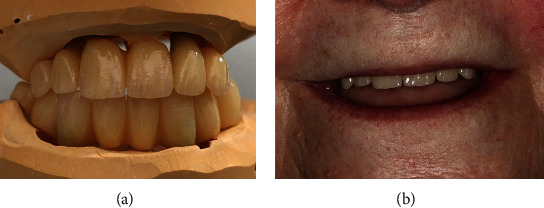
CAD/CAM-milled provisional FPD made out of PMMA (PMMA-Disk, Yamahachi Dental©, Gamagori, Japan) (a). An extreme short dental arch (ESDA) is depicted with an extension from canine to canine (upper jaw) and from first premolar to first premolar (lower jaw). The patient now shows an even smile line and natural visibility of the anterior teeth (b).

To address these challenges, four implants were placed in the lateral incisor positions in both jaws using Straumann© implants (012: 4.1 × 10 mm, 022: 3.3 × 10 mm, 032: 4.1 × 10 mm, and 042: 4.1 × 8 mm; Straumann© Group, Basel, Switzerland) (Figure 2). Implants were placed as distally as possible to cover a larger span of the respective jaw (Figure 3). However, implant positioning and alignment proved to be extremely challenging due to the limited mouth opening, and the handpiece and drill had to be carefully managed to avoid injury. After the implants had healed and osseointegrated, an experimental fixed partial denture (FPD) made of milled PMMA (Yamahachi Dental©, Gamagori, Japan) was fitted (Figure 4).

**Figure 5:**
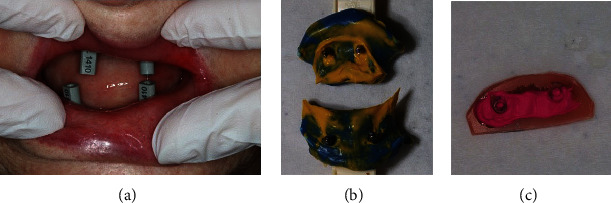
Clinical images were taken at the time of impression taking and registration. Digital impression taking with an intraoral scanner was not possible due to the high mobility of the soft tissues. Scan bodies (L1410, Straumann© Group, Basel, Switzerland) were used as registration aids (a). Bite registration was performed with Beauty Pink wax (Integra Miltex, Princeton, NJ, USA) and Futar® (Kettenbach© Dental, Eschenburg, Germany) in the centric condylar position (c). Severely shortened impression trays with fixed analog impression posts (b). Honigum Putty and Light Body (DMG Dental, Hamburg, Germany) were used as impression material.

However, taking impressions and intraoral scanning proved to be difficult due to the tension of the mucosa and constant movement. To prevent injury and stretching of the mucosa, a digital impression of the implants and soft tissue was attempted but proved unsuccessful. A conventional impression using silicone (Honigum Putty and Light Body, DMG Dental, Hamburg, Germany) and an adapted rigid plastic tray was then taken (Figure 5(b)), which was associated with bleeding at the commissures and severe pain.

The biggest challenge in this case was the bite registration process, as conventional bite rims could not be used due to the patient's limited mouth opening. Instead, an improvised centric registration was taken, similar to that of a patient with full dentition. Scan bodies (L1410, Straumann© Group, Basel, Switzerland) were used as registration aids (Figure 5(a)), as they were readily available, had sufficient height, and provided a flat surface for the wax registration plate to rest on (Figure 5(b)). The wax plate (Beauty Pink, Integra Miltex, Princeton, NJ, USA) was trimmed to size, and an initial centric registration was performed using the chin point guidance technique. The plate was then relined and fitted with silicone (Futar®^,^ Kettenbach© Dental, Eschenburg, Germany) (Figure 5(c)). Additionally, a facebow registration (Artex, Amann Girrbach, Pforzheim, Germany) was performed to adjust the vertical relation in the articulator according to the hinge axis.

The vertical relation was set arbitrarily in the dental laboratory, as it was not possible to determine it in a regular way, and there was no reference to a previous relation available. The corresponding restorations were milled from PMMA and connected to titanium base abutments for screw fixation (Figure 4). A six-month provisional phase followed, during which the patient tested the new occlusal vertical dimension (OVD), occlusion, esthetics, and cleanability of the restorations (Figure 4).

**Figure 6:**
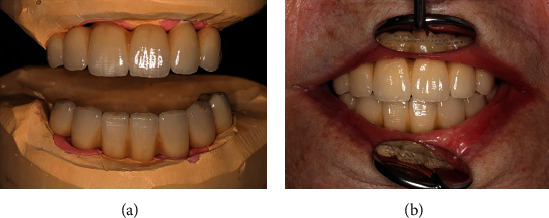
Completed FPD made out of milled and veneered metal-ceramic (Tizian Blank, Schütz Dental, Rösbach vor der Höhe, Germany) (a). Due to the restricted mouth opening, the patient appears completely dentate (b).

Since the patient was satisfied with the provisional restorations and only minimal occlusal adjustments were necessary, the CAD dataset was used to create the final restorations. The FDPs were then screwed into the patient's mouth (Figure 6), with a torque set to 35 N cm in accordance with the manufacturer's instructions. Once again, minimal occlusal adjustment was necessary, and the access holes were sealed with composite (Tetric EvoCeram, Ivoclar Vivadent©, Schaan, Liechtenstein).

The patient returned for a recall appointment one year after the completion of the definitive restorations. During this time, the patient had not visited our policlinic, resulting in the absence of any adverse or unforeseen events documented in their records.

**Figure 7:**
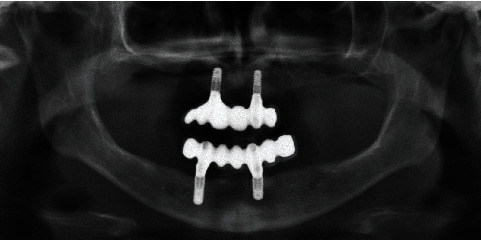
Panoramic radiograph (OPG) taken one year after the placement of the definitive restorations. The framework of the extremely shortened dentition (ESDA) is clearly visible, and the peri-implant bone conditions show no signs of inflammation.

During the recall, we conducted a panoramic radiograph (OPG) (Figure 7) and assessed both intraoral and extraoral examinations. Additionally, we measured probing depths around the implants and conducted a temporomandibular disorder (TMD) screening. The extraoral examination revealed no abnormalities, while intraorally, we detected some calculus in the pontics area, which was promptly removed and polished. The patient displayed excellent oral hygiene overall, with probing depths within the physiological range of 2-3 mm.

The TMD screening yielded no abnormal findings, except for the previously noted mouth opening restriction due to OLS. Notably, the patient continued to report a significant improvement in their quality of life and psychosocial well-being. From the patient's perspective, the therapy measures appear to be a resounding success, despite the absence of posterior teeth.

## 3. Discussion

The restoration of the patient's chewing ability and esthetics not only provided functional benefits but also had a significant positive impact on her psychosocial well-being. According to the World Health Organization (WHO), oral health-related quality of life (OHRQoL) includes the ability to chew and eat food, speak clearly, have a socially acceptable smile, maintain an appropriate dentofacial profile, feel comfortable in the oral area, and be free of pain [15]. The patient's edentulism and symptoms of oral lichen sclerosus (OLS) had severely impaired her OHRQoL and caused significant psychological distress [16]. The restoration of her dentition and oral function not only improved her physical health but also provided a significant boost to her confidence, self-esteem, and social life.

The standard treatment for edentulous jaws would be a complete denture in the maxilla and a two-implant retained overdenture in the mandible. Due to the pronounced pain symptoms, the very limited inter occlusal distance, and the dry oral mucosa, this type of restoration was not feasible in the present case. In addition, access to the oral cavity was severely restricted, especially to the posterior region, because of the rough buccal mucosa. In order to be able to insert and retain dentures, the use of implants was unavoidable. As already mentioned, only the anterior region was suitable. The guidelines for the restoration of edentulous jaws [17] and the McGill and York consensus conferences [18, 19] prescribe a minimum number of four implants in the maxilla and two in the mandible for the restoration of edentulous jaws. However, these values refer to removable prostheses that are retained with attachments.

Therefore, the decision was made to place two implants in each jaw at the position of the lateral incisors to support a fixed partial denture (FPD) restoration. The implant placement was challenging due to the restricted access and limited mouth opening. Nevertheless, proper implant positioning and axis alignment were achieved to ensure stable support for the FPD. The patient was able to successfully test the provisional restoration during the six-month provisional phase and reported improved chewing ability and psychological well-being. The final FPD restorations were fabricated using CAD/CAM technology and securely screwed into place. The authors acknowledge that during the healing phase and prosthetic rehabilitation, there was radiologically evident bone resorption in the area of the maxillary implants. The patient did not exhibit typical clinical signs, and the implants remained stable without mobility. Consequently, considering the patient's advanced age and significant suffering, the decision was made not to remove the implants and to proceed with prosthetic therapy. Revising the implants would have imposed a significant burden on the patient who was already weakened. The subsequent course of action was carried out in agreement with the patient and after providing a detailed explanation. The patient is enrolled in a closely monitored recall system to maintain stability in the peri-implant area and to ensure the longevity of the restorations. After two years, the situation remains stable.

Since this option was excluded in the present case, only fixed restorations with four to six implants would have been possible according to the above guidelines and the Malo concept [20]. Although four implants each were planned preoperatively, only two implants per jaw could have been placed, which were restored with fixed screw-retained bridges [21, 22]. This resulted in an extreme “short dental arch” (SDA) [23], which restored only up to the first premolar or canine. Despite the SDA, the patient did not experience any functional limitations and reported improved chewing ability and psychological well-being. The relationship between the decrease of posterior support and the development of symptomatic temporomandibular disorder (TMD) is still being debated in the literature [24–28]. While current studies suggest that a shortened dentition does not significantly increase the risk of TMD [29], older studies imply that unilateral loss of support zones is particularly unfavorable [30, 31]. An extremely shortened dental arch (ESDA) is defined as the functional level with eight occluding tooth pairs, such as the occlusion of all anterior teeth and first premolars [32]. In Gerodontology, even further reduced tooth levels, such as anterior-only occlusion, are sometimes considered acceptable [32]. Although there are studies opposing the concept of SDA, Manola et al. [33] recommend the concept, especially for medically compromised patients. The concept represents a pragmatic clinical approach aiming at an individual optimum under given conditions. Fueki and Baba [34] reported an improvement in objective masticatory effectiveness with SDAs, although subjective masticatory ability remained unchanged, when using removable or implant-supported dentures. In the present case, the patient had a significant improvement in both objective and subjective masticatory ability, which also increased OHRQoL [35]. This circumstance was certainly due to the fact that the patient had been edentulous for several years and was thus severely limited in masticatory function.

As patients age, their ability to maintain proper oral hygiene tends to decline due to factors such as decreasing eyesight and motoric limitations. It becomes easier for them to clean the easily accessible anterior teeth and premolars than the molars. This is especially crucial in patients with OLS, as the hardened mucous membranes restrict access to the posterior regions to such an extent that adequate hygiene is hardly possible.

## 4. Conclusions

The patient's experimental treatment, which involved only four implants (two implants in each jaw) and the concept of an extremely shortened dental arch, proved to be a satisfactory therapy. Despite the extremely shortened dental arch, the patient was able to recover her masticatory ability, resulting in a significant improvement in her OHRQoL. The treatment also improved the patient's visual appearance, enabling her to participate in social life again. However, it is crucial to emphasize that this prosthetic restoration carries a high risk of failure, primarily due to inadequate oral hygiene and the potential development of peri-implantitis, as evidenced by radiographic bone level changes in the upper implants. The insufficient presence of attached gingiva, in particular, should be carefully considered.

## Data Availability

Data supporting the reported results can be found in the hospital records.
